# Crazy Little Thing Called Sox—New Insights in Oligodendroglial Sox Protein Function

**DOI:** 10.3390/ijms20112713

**Published:** 2019-06-02

**Authors:** Jan Wittstatt, Simone Reiprich, Melanie Küspert

**Affiliations:** Institut für Biochemie, Emil-Fischer-Zentrum, Friedrich-Alexander-Universität Erlangen-Nürnberg, Erlangen, Germany; jan.wittstatt@fau.de (J.W.); simone.reiprich@fau.de (S.R.)

**Keywords:** Sox protein, oligodendrocyte, glia, transcription factor, myelin

## Abstract

In the central nervous system, oligodendrocytes wrap axons with myelin sheaths, which is essential for rapid transfer of electric signals and their trophic support. In oligodendroglia, transcription factors of the Sox protein family are pivotal regulators of a variety of developmental processes. These include specification, proliferation, and migration of oligodendrocyte precursor cells as well as terminal differentiation to mature myelinating oligodendrocytes. Sox proteins are further affected in demyelinating diseases and are involved in remyelination following damage of the central nervous system. Here we summarize and discuss latest findings on transcriptional regulation of Sox proteins, their function, target genes, and interaction with other transcription factors and chromatin remodelers in oligodendroglia with physiological and pathophysiological relevance.

## 1. Introduction

Oligodendrocytes (OLs) are the myelinating cells of the central nervous system (CNS). Proper generation of myelin sheaths and wrapping of neuronal axons ensure rapid signal transduction along neuronal processes. OLs arise through a defined series of developmental steps, including specification of oligodendrocyte precursor cells (OPCs) from neural precursor cells (NPCs), as well as subsequent OPC proliferation and migration to the destined location. In the course of terminal differentiation, mature OLs come into contact with adjacent neuronal axons and form multilayered myelin sheaths that are important for saltatory conduction of electric signals as well as trophic support of axons. Along the different stages of oligodendroglial development, transcriptional regulation is of utmost importance. Transcription factors of the Sox protein family are among the main regulators of specification, proliferation, and migration of OPCs as well as terminal differentiation into mature myelinating OLs and overall myelin maintenance. Their highly conserved high-mobility group (HMG) box domain enables their binding to defined regions of DNA and defines the family. The 20 Sox proteins present in mammals are further subdivided into groups SoxA–SoxH, mainly based on the amino acid identity of their HMG box domain. Since Sox proteins shown to be of highest importance within the oligodendroglial lineage belong to the groups SoxB, SoxC, SoxD, SoxE, and SoxF, this review aims to give an overview of new insights over the last three years into the diverse functional roles of these Sox proteins in oligodendrogenesis, developmental myelination, and de- and remyelination. For further information on the function of Sox proteins in oligodendroglia, please refer to Küspert & Wegner 2016 [1]. For an overview of Sox function across the different cell types of the CNS, refer to Reiprich & Wegner 2015 [2].

## 2. Sox Proteins Regulate Oligodendroglial Specification and Identity

Oligodendroglia of the murine spinal cord arise from the pMN domain, which represents a ventral domain in the ventricular zone of the spinal cord in which motor neurons are generated before the neuron–glia cell fate switch. In this domain, proteins of the SoxE group, which consist of Sox8, Sox9, and Sox10, are expressed in a defined pattern concerning timing and cell specificity. Sox9 is expressed in the ventricular zone of the murine spinal cord from mouse embryonic day 10.5 (E10.5) on, followed by Sox8 in NPCs of the pMN domain. Sox9 represses neurogenesis in NPCs, as studies on Sox9-deficient spinal cord show a transient increase in emergence of neurons. This phenotype is accompanied by a severe defect in the specification of oligodendroglia [3]. Sox8 is also implicated in oligodendroglial specification, although spinal cords of Sox8-deficient mice appear unaltered. However, double-mutant mice lacking Sox8 as well as Sox9 show an increased defect of oligodendroglial specification compared to Sox9 single-mutant mice [4,5]. In contrast to Sox8 and Sox9, Sox10 expression starts only in OPCs after specification.

**Figure 1:**
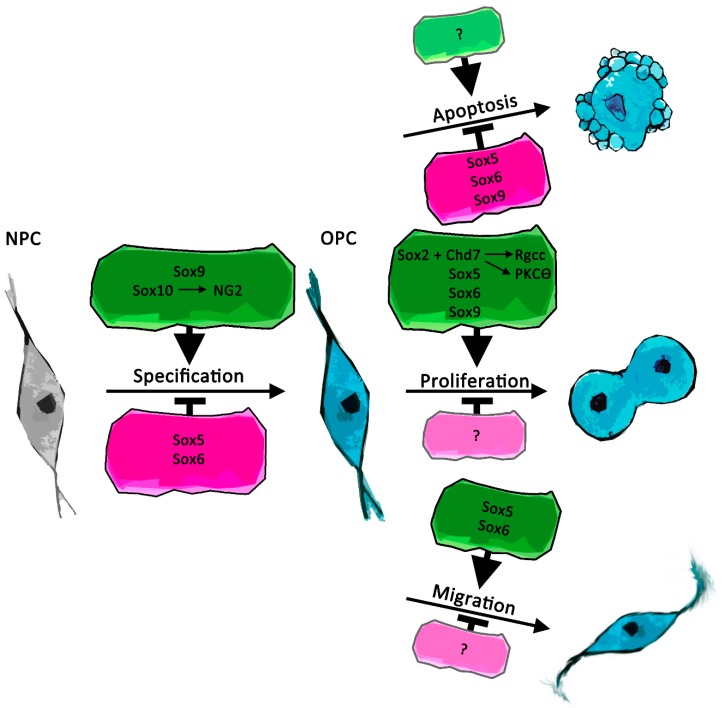
Schematic representation of recently described Sox protein functions during specification, proliferation, and migration of oligodendrocyte precursor cells (OPCs) as well as in regulation of apoptosis. Positive effects are depicted in green, negative effects in magenta. Arrows in colored frames indicate regulators of Sox protein expression as well as mediators of Sox protein function. Interaction partners of Sox proteins are additionally annotated by “+”. NPC = neural precursor cells.

The capability of different SoxE proteins to drive oligodendroglial specification has further been analyzed in a study aiming to generate OLs by expression of a set of transcription factors in human pluripotent stem cell (hPSC)-derived NPCs [6]. NPCs were lentivirally transduced with candidate transcription factors followed by in vitro differentiation in medium containing T3. Cells transduced with either SoxE protein expressed the pan-oligodendroglial marker Olig2 as well as myelination markers Mog, Mbp, and Plp1, while they were negative for the proliferation marker Ki67. Although all three SoxE proteins were able to induce oligodendroglial specification, Sox10 was the most potent driver for the generation of OLs. Noteworthy, while Sox9 is a potent driver of specification in vivo, Sox10 is not involved in developmental OPC specification in mice, as it is not expressed in NPCs prior to the emergence of OPCs. The cells obtained by lentiviral Sox10 overexpression resembled primary intermediate OLs on the transcriptome level. Grafting of these cells into cultured brain slices of homozygous Mbp-deficient shiverer mice led to myelination of neuronal axons proving their ability to behave like native OLs in vivo. Initiation of myelination was further observed in co-cultures with cortical neurons derived from human induced PSCs. Previous reports stated a set of three transcription factors—Olig2 and Sox10 combined with either Zfp536 or Nkx6.2—to be necessary for the generation of induced OPCs from mouse as well as rat fibroblasts [7,8], whereas sole Sox10 addition only resulted in a minor OPC yield [7]. OPCs obtained by a combination of the aforementioned three transcription factors were likewise capable of differentiating into myelin protein-expressing OLs. By already choosing NPCs as starting point for their assay, Garcia-Leon and colleagues probably reduced the factors necessary for OPC induction and, concurrently, emphasized the prominent role of SoxE factors in the oligodendroglial lineage decision (Figure 1).

Furthermore, the importance of Sox10 for oligodendroglial identity has recently been confirmed, as it was shown to bind and transactivate regulatory regions of NG2, a proteoglycan encoded by the *Cspg4* gene and expressed in embryonic and adult OPCs [9,10]. NG2 is expressed shortly after OPC specification immediately following Sox10, is downregulated upon terminal differentiation, and regulates cell adhesion, migration, and proliferation [11,12]. Gotoh and colleagues have identified a new Sox10-dependent enhancer within the first intron of the *Cspg4* gene. This was identified by *in ovo*-electroporation of an EGFP-reporter construct under the control of this newly identified enhancer in chick spinal cord as well as by transfection in mixed glial cells derived from perinatal mouse brain which predominantly labelled OLs. Analysis of transgenic mice carrying a similar reporter construct revealed that the enhancer was active in OPCs of the neocortex but not in vascular pericytes, another cell type expressing NG2 in the murine CNS. Furthermore, chromatin immunoprecipitation (ChIP) experiments identified binding of Sox10 to this enhancer region. Additional electromobility shift assays (EMSAs) and luciferase reporter gene assays confirmed binding of Sox10 and showed its transactivation capacity. Consistently, mutation of the comprised Sox consensus motifs led to reduced transactivation by Sox10 and provided supporting evidence for Sox10 as an activator of NG2 expression in oligodendroglial cells (Figure 1). Sox10 furthermore activated the enhancer in a cooperative manner together with the bHLH transcription factors Ascl1 or Olig2 and E47.

## 3. Proteins of Different Sox Groups Interact during Oligodendroglial Specification

At E11.5, just around the onset of oligodendroglial specification in the mouse spinal cord, the expression of Sox9 overlaps with the expression of SoxB1 proteins in NPCs [3,13]. The family of SoxB1 proteins consists of Sox1, Sox2, and Sox3, which are coexpressed in most NPCs, and Sox2 is widely regarded as a stemness and pluripotency factor with a pivotal role in regulating proliferation and specification of NPCs. Furthermore, previous reports state that Sox2 and Sox3 in NPCs prebind regulatory regions associated with neuronal gene expression, which are transcribed only later after specification of neurons [14].

A recent study aimed to investigate the effect of Sox9 on oligodendroglial specification and its possible interplay with Sox3 when prebinding regulatory gene regions in NPCs [15]. Single-cell RNA sequencing data from CD133-sorted spinal cord NPCs of E11.5 old mice, as well as from cells isolated from selected regions of the E15.5 mouse spinal cord tissue, were obtained. These datasets were combined with ChIP-seq data of Sox9 and Sox3 genomic occupancy and H3K27 acetylation in NPCs and glial precursor cells derived from embryonic stem cell cultures. A model was established in which Sox3 prebound regulatory sites of neuronal and glial genes in NPCs. According to this model, Sox3 colocalized at many Sox9-bound sites in the proximity of astrocytic genes, while genes related to the oligodendroglial lineage showed sole binding of Sox9. Considering previously published Sox10-ChIP-seq data, Sox10 predominantly targeted sites at glial genes prebound by Sox9 in the absence of Sox3. Reporter gene assays with Sox9/Sox10 bound sites around the OL-specific genes *Plekhb-1* and *Mid2* showed additive activation of reporter genes by Sox10 and Sox9, further confirming the importance of SoxE protein interplay in the consolidation of oligodendroglial identity (Figure 1).

## 4. Sox Proteins Regulate Developmental Characteristics of Oligodendrocyte Precursor Cells

**Figure 2:**
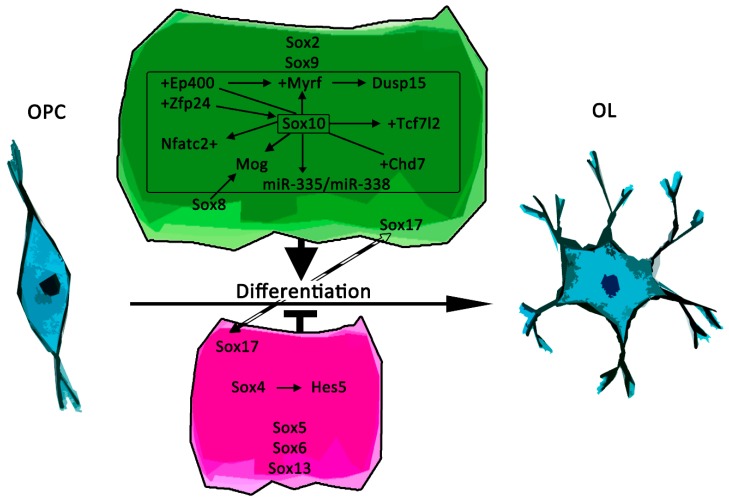
Schematic representation of recently described Sox protein functions in oligodendroglial differentiation. Positive effects are shown in green, negative effects in magenta. Arrows in colored frames indicate transcriptional regulators and mediators of Sox protein function, while proteins that directly interact with Sox proteins are further annotated by “+”. Dashed arrow between negative and positive regulation indicates switch of Sox protein function. OL = oligodendrocyte.

While most of the findings on OPC specification have been obtained from experiments on the spinal cord and the forebrain, the origin of cerebellar OLs and their proliferation capabilities have only recently been addressed [16]. The majority of OLs in the cerebellum were derived from Olig2 expressing cells of the neuroepithelial domain of ventral rhombomere 1 (vr1), as determined by in vivo fate mapping in combination with immunohistochemical stainings for different stage-specific markers of the oligodendroglial lineage. An additional small fraction of cerebellar OLs originated in the cerebellar ventricular zone. Previous studies have shown that Sox9 and Sox10 are involved in the regulation of proliferation, survival, and migration of spinal cord OPCs, for instance by regulating the expression of Pdgfra [17]. Conditional deletion of Sox9 in the cerebellum, vr1 region, and caudal midbrain using En1::Cre revealed the importance of this SoxE protein for the development of already specified cerebellar OLs in the mouse. While the analysis of neuronal and astrocytic markers exhibited no difference, oligodendroglia were reduced in numbers (from E16.5 on) when compared to control mice. This reduction was attributed to a decrease in proliferation of OPCs as well as an increase in apoptosis in the cerebellum (Figure 1). The observed reduction of cerebellar oligodendroglia in the absence of Sox9 is consistent with previous analyses in the murine spinal cord, where deletion of Sox9 in Nestin-positive cells led to a drastic reduction of OPCs from E12.5 on [3]. Unlike in the En1-positive area, the reduction of spinal cord OPCs is attributed to disturbed oligodendroglial specification. In fact, OPC proliferation is elevated in the spinal cord, resulting in partial recovery of OPC numbers at E16.5 compared to controls. However, similar to OPCs of the En1-positive area, an overall increase of apoptosis at E18.5 in the spinal cord accompanied by general tissue degeneration was shown. In the recent publication, loss of Sox9 furthermore led to a diminished arborization of mature OLs as well as thinner myelin sheaths, as observed in electron microscopy images. Immunocytochemical stainings of differentiated OLs derived from Sox9-deficient oligospheres additionally confirmed the effect on OL maturation as seen by reduced immunoreactivity for Mbp under differentiation conditions (Figure 2). Hashimoto et al. thereby showed that while loss of Sox9 did not affect the initiation of oligodendroglial differentiation in the spinal cord as a result of compensatory functions of Sox10 [17], deletion of Sox9 was sufficient to impair the development of OLs in the En1-positive area in the mouse brain.

Besides SoxE proteins, members of the SoxD protein group also affect oligodendroglial specification as well as terminal differentiation. The SoxD group is composed of Sox5, Sox6, and Sox13. Sox5 and Sox6 are expressed in NPCs of the spinal cord as well as in OPCs. In the oligodendroglial lineage, SoxD proteins counteract SoxE protein function by competing for shared DNA binding sites or interaction partners without acting as transcriptional activators on their own, as they lack a transactivation domain. Combined constitutive deletion of Sox5 and Sox6 in the spinal cord leads to precocious specification of OPCs, migration defects, and premature expression of maturation markers such as Plp-1 and Mbp [18]. A recent publication of Baroti and colleagues now confirmed these defects after conditional deletion of Sox5 and Sox6 in the CNS and an expanded the analysis to the murine forebrain [19]. Migratory defects in the spinal cord were validated as migrating OPCs in Sox5- and Sox6-deficient spinal cords were first observed at E15.5, while wild-type OPCs had already started to migrate out of the pMN domain at the earliest time of analysis at E13.5. OPCs were not dispersed over the whole spinal cord at E15.5 but were restricted to the ventral half where they were generated. Furthermore, Pdgfra-positive OPCs were reduced (Figure 1), and OPCs additionally displayed premature expression of myelin genes (Figure 2). As the observed phenotype in Sox6 single-mutant mice was milder, it was assumed that Sox5 and Sox6 compensated for the loss of each other in migration and proliferation of OPCs as well as in oligodendroglial differentiation. Furthermore, analysis of OPCs of embryonic forebrains in Sox5/Sox6 conditional double-knockout mice displayed increased apoptosis and reduced proliferation, resulting in reduced numbers of Sox10-positive oligodendroglia in the mantle zone. Forebrain OPCs also exhibited migratory defects and premature maturation of progenitors. Noteworthy, apoptosis in the spinal cord and forebrain was increased at postnatal stages (Figure 1). Therefore, it was hypothesized that prematurely differentiated OPCs died as a result of their altered distribution by programmed cell death caused by the absence of accessible axons in their vicinity, which is needed for long-term OL survival. The expression pattern of the third SoxD member Sox13 resembled the pattern of Sox5 and Sox6 [20]. Numbers and temporal appearances of Sox10-positive cells in the pMN domain of Sox13-knockout mice were not altered in the spinal cord at any analyzed embryonic stage. The additional deletion of Sox13 in Sox6-deficient OPCs did not lead to a more severe phenotype of proliferation and migration. However, during terminal differentiation, an increase in Myrf- and Mbp-positive cells in the double-mutant mice compared to the Sox6 single-mutant indicated a function of Sox13 as negative regulator of terminal differentiation (Figure 2). Gel retardation assays and reporter gene assays revealed a repressive function of Sox13 on Sox10-dependent activation of the *Mbp* promoter. Therefore, Sox13 showed some functional redundancy with Sox5 and Sox6 with only minor functions during OL development.

## 5. Sox Proteins Influence Terminal Differentiation

The SoxC proteins Sox4 and Sox11 are known to act as inhibitors of OL differentiation. Both are highly expressed in OPCs and are downregulated during terminal differentiation, while prolonged expression of Sox4 in OLs results in a hypomyelination phenotype [21].

Recent experiments of shRNA-mediated knockdown of Sox4 in neural stem cells (NSCs) further clarified the functional role of SoxC proteins in maturation of OLs [22]. Sox4-knockdown resulted in an increased number of CNPase-positive mature OLs when kept for five days in oligodendroglial differentiation conditions, while specification of OPCs was not disturbed. Compatible with the phenotype seen after prolonged Sox4 expression in vivo, overexpression of Sox4 also in vitro resulted in reduced OPC differentiation. As a mediator of Sox4 effects, the authors identified the bHLH protein Hes5. It was shown to be a direct target of Sox4 by comparing Sox4-ChIP-seq data of NSCs with RNA-seq data of Sox4-knockdown NSCs. Hes5 is an effector of the Notch pathway, which was previously shown to be involved in negative regulation of oligodendroglial differentiation [23,24]. In addition, Braccioli and colleagues could confirm that overexpression of Hes5 in NSCs was sufficient to revert the Sox4-knockdown phenotype of enhanced OL maturation (Figure 2).

Proteins of the SoxB1 family for a long time were only known for their important roles in stem cell maintenance and, therefore, as inhibitors of specification in the CNS [25,26,27]. Only in the last few years, new functions for SoxB1 factors in the regulation of terminal OL differentiation were identified. Studies on mice with combined deletion of Sox2 and Sox3 in Sox10-positive cells indicate that neither protein is necessary for OPC migration or proliferation in the embryonic spinal cord. But reduced numbers of Mbp- and Plp-expressing cells at E18.5 point to a delay in initiation of OL differentiation [28]. As Sox2 and Sox3 double-deficient mice died around birth, single mutants for Sox2 or Sox3 in Sox10-positive cells were used to show that reduced myelin gene expression persisted into early postnatal stages. Reporter gene assays confirmed that Sox2 activated expression from the *Mbp* promoter, although it was less effective than Sox10. Furthermore, Sox2 derepresses factors important for differentiation by repressing miR-145, a microRNA that inhibits the promyelinating factors Myrf and Med12.

Recent research has shown that mice with a deletion of Sox2 in Sox10-positive cells developed tremors and ataxia 28 days after birth with impaired motor coordination as assessed by rotarod experiments. Analysis of 60 days old animals showed reduced density of myelinated axons in the corticospinal tract. Already at P19, diminished expression of pan-oligodendroglial genes and a decrease in Cnp1 as well as Mbp protein were shown in the spinal cord compared to controls [29]. In agreement with the previous study on Sox2 function in OLs [28], the authors did not see a reduction of overall OPC numbers in the embryonic spinal cord of these mice. They further performed tamoxifen-induced early postnatal deletion of Sox2 in Pdgfra-positive precursor cells to test whether the reduction of the postnatal OPC population was an accumulative effect of embryonic Sox2-deletion or an immanent effect of postnatal Sox2-deficiency. Interestingly, a reduction of OPC numbers was observed at P8 and P26 and was attributed to impaired proliferation, as Ki67-positive cells and EdU incorporation were reduced, while apoptosis was not altered at P8. This indicates a functional role of Sox2 in postnatal OPC proliferation (Figure 1). Furthermore, the analysis of several myelin-markers showed a reduction of maturity factors on mRNA levels. Stainings against Tcf7l2, which marks newly formed OLs, on spinal cords of mice with deletion of Sox2 in Cnp1-positive cells at P14 provided additional evidence for the importance of Sox2 in OL differentiation (Figure 2), as fewer newly formed OLs were observed. Zhang et al. could also show increased Sox2 fluorescence intensity in Tcf7l2-positive cells cultured from forebrain tissue as well as in the forebrain corpus callosum at P8 and P14. Deletion of Sox2 in Cnp1-positive cells likewise impaired myelination in the subcortical white matter, resulting in a hypomyelination phenotype as seen by reduced Mbp protein levels and a reduction of CC1-positive cells. As in the spinal cord, the total number of OPCs was reduced in the forebrain at P8 and P14 in mice with postnatal deletion of Sox2 by tamoxifen induction in Pdgfra-positive cells at P6 and P7 [30].

The most prominent member of the SoxF protein group with reported (although only minor) function in oligodendroglial development is Sox17, which is expressed at its highest just before the onset of terminal differentiation. It interferes with Wnt/ß-catenin signaling at the initiation of OL differentiation. Sox17 promotes the expression of myelin genes and cell cycle exit by affecting the induction of the Wnt downstream effector CyclinD1 in vitro [31,32]. Moreover, overexpression of Sox17 under control of the Cnp1 promoter increases OL differentiation in subcortical white matter of adult mice [33].

A recent approach of Sox17 overexpression in Sox10-positive cells showed that excess Sox17 decreased the number of cells that were positive for the maturation marker CC1 at P3 and P7 in the spinal cord but not at the time of birth or at P15 [34]. As proliferation of OPCs at E15.5 and the total number of OLs in the murine spinal cord were unchanged even at early postnatal stages, it was assumed that Sox17 overexpression led to a temporary delay in OL maturation. This observation was confirmed by reduced myelin thickness and decreased numbers of myelinated axons at P7 when compared with control mice. Downregulation of Enpp6, a marker specific for newly formed OLs, at P3 further strengthened the hypothesis of Sox17 as a regulator of transition from OPCs to newly formed OLs. The transient delay of OL differentiation in the spinal cord contrasts previous findings of increased differentiation in adult white matter after Sox17 overexpression [33]. Fauveau and colleagues attributed these discrepancies to different timing and dynamics of Sox17 overexpression. As Sox17 overexpression beginning with the OPC stage resulted in temporary reduction of mature OLs, while late Sox17 overexpression increased differentiation, Sox17 was speculated to switch from negative to positive regulation of differentiation along OL development (Figure 2).

## 6. Sox Proteins Can Regulate Each Other during Initiation of Terminal Differentiation

In contrast to SoxF proteins, SoxE proteins are major regulators of terminal OL differentiation. For instance, conditional deletion of Sox10 results in a strong hypomyelination phenotype represented by a dramatic decrease in the number of myelin sheaths as well as by reduced expression of myelination markers. Sox10 partially acts by directly inducing structural myelin proteins such as Mbp. Aside from positive regulation of myelin-related genes, Sox10 indirectly inhibits factors associated with the progenitor state including Sox9 [35]. Being coexpressed in the early OPC stage, levels of Sox10 increase after initiation of terminal differentiation, while Sox9 gets successively lost [3,36]. After the onset of Sox10 expression, Sox9 is downregulated in late OPCs, and its reduction appears to be a prerequisite for the differentiation into mature OLs. Deletion of Sox10 in an oligodendroglial cell line in turn resulted in an increase in *Sox9* transcription. In silico analyses combined with an miRNA-screening approach identified two miRNAs, *miR-335* and *miR-338,* as Sox10-regulated genes that were involved in the posttranscriptional regulation of Sox9 expression. EMSAs and luciferase reporter gene assays confirmed binding and activation of *miR-335* and *miR-338* regulatory regions by Sox10. Reporter gene assays proved negative regulation of Sox9 expression through binding of these miRNAs to the *Sox9* 3′-UTR and thereby supported the hypothesis of a negative regulatory circuit predicted by mathematical modelling. This feedback inhibition of Sox9 expression by Sox10 may constitute an irreversible switch shifting the oligodendroglial expression pattern and thereby promoting the process of OL differentiation (Figure 2).

## 7. Sox10 Interacts with Multiple Cofactors in the Regulation of Terminal Differentiation

Sox10 induces not only expression of structural myelin proteins but also regulatory proteins such as the transcription factor Myrf, which is essential for terminal differentiation [37]. Binding of Sox10 to regulatory regions of myelin genes in many cases occurs in concert with additional regulatory factors. They modulate Sox10 function by conveying temporal or spatial specificity for target genes or boosting Sox10-mediated activation for rapid induction of myelin genes. In OPCs, Sox10 expression is dependent on the bHLH transcription factor Olig2 [38]. A recent report identified Zfp24 as a regulator of Sox10 transcription and then as a cofactor of Sox10 in the induction of Myrf expression [39]. The authors first showed that Zfp24 in OPCs was predominantly present in a phosphorylated state, which prohibited binding to DNA. In more mature OLs, Zfp24 is dephosphorylated, thereby acquiring the ability to bind to DNA. Zfp24-ChIP-seq data obtained from primary mouse OPCs and mature OLs identified Zfp24 consensus-binding motifs upstream of the transcriptional start site of *Sox10*. Direct Zfp24 binding to this region was confirmed by EMSAs. In reporter gene assays, Zfp24 was also able to transactivate reporter gene expression under the control of this regulatory region, further indicating its functional relevance. Comparison of RNA-seq data of Zfp24-null mutant OLs with Zfp24-ChIP-seq data additionally revealed that Zfp24 indirectly regulated the expression of myelin genes via direct control of Myrf and Sox10 expression. Myrf has previously been shown to be induced by Sox10 and to cooperate with its inducer in the activation of myelin genes [37]. Interestingly, Elbaz and colleagues found shared DNA binding sites for Zfp24 with Myrf, Sox10, and Olig2 upstream of genes essential for OL maturation, which pointed to joint activation of those genes by Zfp24 together with several key regulators of myelination (Figure 2).

Recently, the dual specificity phosphatase Dusp15 was identified as a target gene of Sox10 and Myrf with an influence on OL differentiation [40]. Dusp15 expression was restricted to differentiating OLs and shRNA-mediated knockdown in primary rat OPCs resulted in a reduction of Mbp expression compared to control OLs, while retroviral overexpression transiently increased the number of Mbp-expressing OLs. Dusp15 transcripts were shown to be severely decreased in spinal cords of P7 mice with a CNS-specific knockout of Sox10, while overexpression of Sox10 increased Dusp15 expression at E18.5. Furthermore, luciferase reporter gene assays and EMSAs showed joint activation of the *Dusp15* promoter by Sox10 and Myrf, and they identified Dusp15 as a novel direct target of both regulators during OL differentiation as well as a mediator of their prodifferentiative functions (Figure 2).

The HMG box transcription factor Tcf7l2 was also recently identified as one of the cofactors working in concert with Sox10 during OL lineage progression [41]. Tcf7l2 is already described as the major transducer and transcriptional partner of ß-catenin in the Wnt signaling pathway, and it is temporarily expressed at high levels in premyelinating OLs. This led to the hypothesis that Tcf7l2 acts as a repressor of OL differentiation through activation of Wnt target genes [42,43]. Recent work from Zhao and colleagues in contrast showed that loss of Tcf7l2 in the mouse blocked differentiation of OLs, as seen by a postnatal reduction in myelin marker expression in the cortex and a reduced number of myelinated axons in the corpus callosum at P14 and P60 in mice. Mbp levels and myelin thickness in the spinal cord were likewise affected at P0 and P7, while myelination recovered until P60. Comparison of ChIP data obtained for Sox10 and Tcf7l2 showed enriched binding of Sox10 at Tcf7l2-bound sites in maturing OLs and co-immunoprecipitation experiments verified direct interactions of Sox10 and Tcf7l2 on protein level (Figure 2). Functional interactions of Sox10 and Tcf7l2 during OL maturation were shown for the activation of mature myelin proteins as well as cholesterol synthesis genes in reporter gene assays.

To identify more of these regulatory interactions necessary for proper OL differentiation and myelination, Cantone and colleagues used bioinformatics and meta-analyses of high-throughput data [44]. They combined experimentally validated and computationally predicted interactions in an interactive gene regulatory network web tool and experimentally verified some of the predicted interactions. Interestingly they showed that Tcf7l2 expression was induced by Sox10 via binding to *Tcf7l2* regulatory regions but later was downregulated by two miRNAs, miR-155 and miR-338, that were both direct targets of Sox10. Therefore, Sox10 is a major regulator of transient Tcf7l2 expression and interacts with it for proper timing of myelin gene expression during myelination (Figure 2).

In a recent publication, another interaction partner for Sox10 was identified with Nfatc2 [45]. Nfat proteins are activated by the calcium-dependent phosphatase Calcineurin. Thus, they may enable integration of external stimuli in OL differentiation such as contact to actively firing neuronal axons, which can induce myelination via promotion of Ca^2+^ influx into OPCs [46,47]. Weider et al. now could show that Nfatc2 is a direct target of Sox10 and additionally interacts with it in terminal OL differentiation. Nfat signaling was proven to be important for the initiation of OL differentiation in vitro and in vivo. Functional relevance of Nfat signaling was assessed in primary rat OPCs by using a blocker of Calcineurin-dependent activation of Nfats as well as in mice carrying a deletion of Calcineurin mediated by *Sox10*- or *Cnp1*-driven Cre expression. Blocked Calcineurin signaling as well as Calcineurin-deficiency in mice led to a reduced expression of several OL differentiation markers, whereas proliferation and apoptosis remained unchanged. Expression of Nfatc2 was reduced in Sox10-deficient oligodendroglial cells in vitro, and lentiviral expression of Sox10 was sufficient to restore its expression. Further analysis by EMSAs and luciferase reporter assays confirmed the effects of Sox10 on Nfatc2 expression. While Nfatc2 alone was not able to induce reporter gene activity under the control of myelin gene regulatory elements or to enhance Sox10-dependent stimulation of these regulatory regions, it was shown to increase the transactivation capability of Sox10 at *Nkx2.2* evolutionary conserved regions. *Nkx2.2* is known to be important in the timing of terminal differentiation and to directly regulate expression of several myelin genes. Nfatc2 binding to regulatory regions of *Nkx2.2* was confirmed in EMSAs, and joint activation by Nfatc2 and Sox10 was verified by electroporation in the chicken neural tube at Hamburger–Hamilton stage 11, thereby identifying Calcineurin/Nfat-signaling as an important regulatory module during OL differentiation (Figure 2).

During myelination, chromatin is heavily reorganized to allow transcription of myelin genes. The TIP60/EP400 complex, a chromatin remodeling complex of the INO80/SWR subfamily, was recently identified to interact with Sox10 during oligodendroglial development [48]. Deletion of Ep400, which is the ATP-hydrolyzing subunit of this chromatin remodeling complex, in Nestin-positive NSCsshowed that it is dispensable for OPC specification, proliferation, and migration in the murine embryonic spinal cord. Ep400-deletion in Sox10-positive cells was further used to study its cell-autonomous function in the oligodendroglial lineage. While proliferation and migration of OPC remained unaltered, the initiation of terminal differentiation was drastically impaired as assessed by reduction of Mbp- and Myrf-positive cells in the spinal cord at E18.5. Hypomyelination was also observed in the spinal cord and forebrain of mice with an Ep400-deletion in Cnp1-positive late OPCs, and it persisted throughout all analyzed postnatal stages. Ultrastructural analyses at P21, P32, and P60 revealed a reduced number of myelinated, large-caliber axons and reduced myelin thickness in the spinal cord of mutant mice. The expression profile of oligodendroglia with Ep400-deletion in Cnp1-positive cells as well as ChIP experiments and reporter gene assays demonstrated that Ep400 regulated the expression of Myrf by binding to its enhancer ECR9. As it is known that Sox10 also binds to ECR9, the authors confirmed the interaction of Sox10 and Ep400 by co-immunoprecipitation experiments (Figure 2). In additional pulldown experiments, Sox10 was found to interact with Ep400 via its dimerization and HMG-domain, whereas Sox10 bound the HSA and the SANT domains of Ep400. These findings suggest that Ep400 and Sox10 cooperate in the activation of *Myrf* at ECR9 during OL differentiation.

## 8. Sox Proteins Are Important for Myelin Maintenance

Proteins of the SoxE family partially fulfill redundant functions in oligodendroglia because they have similar structures. For example, Sox10-deficient mice do not show a complete loss of myelin gene expression. Residual myelin gene expression is attributed to the closely related transcription factor Sox8, although compensation capabilities of Sox8 are only partial [5]. This is also seen in a mouse model where the open reading frame of Sox10 was replaced by that of Sox8. In these mice, the number of myelinating OLs was not completely restored, but they were still severely impaired [49].

**Figure 3:**
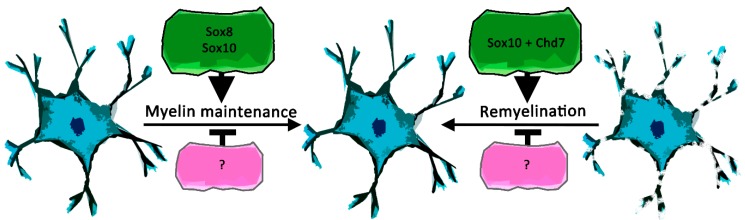
Schematic representation of recently described Sox protein functions in oligodendroglial myelin maintenance and remyelination. Positive effects are shown in green, negative effects in magenta, while proteins that directly interact with Sox proteins are annotated by “+”.

In a recent report, Turnescu and colleagues aimed to elucidate the role of Sox8 in OLs and found a more decisive function in myelin maintenance [50]. In mice with a late deletion of Sox10 in the final stage of differentiation in Mog-expressing OLs, myelin markers or the overall numbers of oligodendroglia were not affected. Instead, a slight upregulation of Sox8 expression was observed on transcript level. Comparably, a single knockout of Sox8 had no significant effect on marker gene expression in OPCs and mature OLs at P30, P60, and P100. In contrast, late deletion of Sox10 in the background of a constitutive Sox8-knockout led to a shivering phenotype starting at around P21. These mice showed reduced numbers of cells that expressed maturation markers Myrf, Mbp, and Plp1. Fewer and thinner myelin sheaths were detectable, as well as secondary axonal degradation, in the double-knockout spinal cords when compared to controls. Moreover, EMSAs and luciferase assays identified *Mog* as a combined target of Sox8 and Sox10 (Figure 2), which directly linked both SoxE factors to the regulation of late terminal differentiation, therebyidentifying a new auxiliary role of Sox8 during myelin maintenance (Figure 3).

## 9. Sox Proteins Are Implicated in Myelin-Related Diseases and Disease Models

Besides their importance for OL development during embryogenesis and in early postnatal stages, regulatory functions of Sox proteins have been implicated in diseases with CNS pathology such as the PCWH syndrome in the case of Sox10 mutations [51]. Recent work on oligodendroglial development under disease conditions stressed the connection of the chromatin remodeler Chd7 with Sox proteins [52]. Chd7 is responsible for the spatiotemporally correct expression of several developmental genes and mutations, and Chd7 accounts for the majority of human CHARGE syndrome cases. Manifestations of the CHARGE syndrome exhibit a variety of congenital defects including deafness, retarded growth, heart defects, and CNS anomalies [53,54,55].

For instance, MRI scans of patients with CHARGE syndrome revealed abnormalities in the cerebellar white matter with volumetric loss and dysmorphic features in 47% of cases [56]. As interaction studies in an oligodendroglial cell line showed protein binding of Sox10 to Chd7 under differentiation conditions, mice with a deletion of Chd7 in Olig1-positive cells were used to study the interplay of Chd7 and Sox10 in the oligodendroglial population. The loss of Chd7 led to a reduction of pre- and postnatal Mbp- and CC1-positive cells. The overall pool of oligodendroglia was reduced as determined by stainings against Sox10, Olig2, and Olig1, while no defects in OPC proliferation nor reduced numbers of OPCs were observed in cortices at P7 and P14. Although myelin morphology and the number of myelinated axons recovered in 8-week-old Chd7 conditional knockout animals, remyelination after lysolecithin-induced demyelination was impaired in the spinal cord and corpus callosum. ChIP-seq experiments revealed that Sox10 and Chd7 jointly targeted a common set of myelin-related genes such as *Myrf*, *Nkx2.2,* and *Mbp*. Reporter gene assays showed a cooperative transactivation capability on promoter regions of *Plp1* and *Cnp1*, which further indicated functional relevance of the Chd7-Sox10 interaction in oligodendroglial development, myelination, and remyelination (Figure 2 and Figure 3).

Marie and colleagues further compared ChIP-seq data sets of Sox10, Olig2, and Chd7 from cortices of P7 mice, and they found only a fraction of jointly targeted oligodendroglial genes were deregulated upon Chd7 depletion [57]. Hence, additional ChIP-seq for the related Chd8 was performed to confirm co-binding of Chd7 and Chd8 at genomic loci of known regulators of OL development. The results strengthened the hypothesis that Chd8 was partially able to compensate for the loss of Chd7 in OL development. Oligodendroglial cells with tamoxifen-induced deletion of Chd7 in Pdgfra-positive cells showed reduced expressions of Sox10, Nkx2.2, Tcf7l2, and Gpr17. This indicated a role of Chd7 in controlling the timing of OL differentiation in part by regulating the expression of and interacting with Sox10 during the initiation of terminal differentiation (Figure 2).

Chd7 has not only been functionally implied in OL maturation but also recently in proliferation and maintenance of oligodendroglial progenitor cells [58]. Chd7 is co-expressed with the SoxB1 protein Sox2, and an increase in Chd7/Sox2 double-positive OPCs was found after contusive spinal cord injury. Tamoxifen-induced ablation of Chd7 in Pdgfra-positive OPCs led to reduced proliferation that resulted in reduction of mature OLs as well as disruption of myelin structures. Interestingly, these effects phenocopy the proliferation defects observed in retroviral Sox2-knockdown in vitro experiments. These knockdown experiments confirm the finding of perturbed OPC proliferation in the postnatal Sox2-deficient spinal cord described above, while in vitro proliferation assays proved unsuitable to recapitulate the phenotype of Sox2-deletion in the embryonic spinal cord [28,29]. Since, in recent study, combined ablation of Chd7 and Sox2 did not aggravate the respective single phenotypes on OPC proliferation, it is plausible that both proteins act in the same signaling cascade. The hypothesis of joint action was reinforced by co-immunoprecipitations and proximity ligation assays, which confirmed interaction of both proteins in OPCs. ChIP analysis further suggested that the effects on OPC proliferation were partially mediated by the activation of the proteins Rgcc and PKCϴ, which both are implied in cell cycle progression in other cell types. In fact, functional experiments confirmed that knockdown of either of these effectors was able to phenocopy Sox2/Chd7 ablation (Figure 1). As no change in OPC proliferation was previously described for Chd7-deletion [56], Doi and colleagues discussed the different Chd7-deletion strategies as a possible cause of this discrepancy. As these studies differ only slightly in timing of Chd7-deletion, and another recent study did not find a defect in OPC proliferation in Chd7-deficient spinal cord [57], the question of whether Chd7 is a regulator of OPC proliferation remains to be resolved.

## 10. Final Remarks

While recent publications did not identify Sox family members other than the previously known to be involved in OL development, several new functions, interaction partners of Sox proteins, and mediators of Sox protein function were discovered in the oligodendroglial lineage (Table 1). For instance, recent studies proved positive roles for SoxB1 proteins in the maturation of OLs. Finding further interaction partners such as Tcf7l2 and chromatin remodeler subunit Ep400 in OL differentiation, as well as the identification of Sox protein target genes, deepened our understanding of their involvement in regulatory circuits important for oligodendroglial development. Interaction and dynamics of Sox proteins belonging to the same family were elucidated in several oligodendroglial developmental steps, thereby showing redundant as well as unique functions. For instance, SoxD proteins Sox5 and Sox6, showed redundant functions in oligodendroglial development, while the SoxE group members Sox9 and Sox10 played essential roles that were partly shared and partly unique. Sox10, for instance, indirectly inhibits Sox9 during the initiation of terminal differentiation in the spinal cord, and both partially fulfill antagonistic functions during this process. Sox9 function in terminal differentiation in the En1-positive area of the brain is, in part, comparable to the function of Sox10 in the spinal cord. Recent implications in demyelinating disease models additionally emphasized Sox function in cooperation with chromatin remodelers such as Chd7 in CNS development and remyelination. Future research should address the exact mechanisms of Sox protein function in several cases. Until now, for instance, how Sox17 switches from a promoter to an inhibitor of oligodendroglial differentiation is still unresolved. Furthermore, the reason for the varying impact of Sox9 on the initiation of OL maturation in En1-positive areas and the spinal cord require further investigation. In addition, the differences in the proliferative role of Sox2 in OPCs between pre- and postnatal development are yet to be clarified. As oligodendroglia undergo large changes in the transcriptional landscape during differentiation, including the silencing of genes related to the precursor state and making maturation genes accessible for transcription, the identification of novel functions of Sox proteins, and especially interactions with additional chromatin remodelers, remains a tempting field of research. Taken together, the findings of recent publications underline the central role of Sox proteins in the oligodendroglial lineage and broaden our understanding of the OL regulatory network, while they direct future research to promising and, likewise, challenging tasks.

## Figures and Tables

**Table 1:** Summary of oligodendroglial Sox protein functions described in this review. Sox proteins are ordered according to corresponding Sox groups and reported protein functions are listed together with known interaction partners as well as target genes.

Sox Group	Sox Protein	Oligodendroglial Function	Interaction Partner	Target Gene	Reference
**SoxB1**	**Sox2**	Positive regulator of postnatal OPC proliferation	Chd7	*Rgcc, PKCϴ*	[29,30,58]
		Positive regulator of OL differentiation		*Mbp, miR-145*	[28,29,30]
	**Sox3**	Positive regulator of OL differentiation			[28]
**SoxC**	**Sox4**	Negative regulator of OL differentiation		*Hes5*	[21,22]
**SoxD**	**Sox5**	Positive regulator of OPC migration			[18,19]
		Negative regulator of OPC specification, apoptosis and OL differentiation			[18,19]
	**Sox6**	Positive regulator of OPC migration			[18,19]
		Negative regulator of OPC specification, apoptosis and OL differentiation			[18,19]
	**Sox13**	Negative regulator of OL differentiation			[20]
**SoxE**	**Sox8**	Positive regulator of OL differentiation		*Mog*	[50]
		Positive regulator of OL myelin maintenance			[50]
		Positive regulator of OPC specification			[5]
	**Sox9**	Regulated by miR-335 and miR-338			[35]
		Positive regulator of OPC specification			[3,15]
		Positive regulator of OPC proliferation and migration		*Pdgfra*	[3,16,17]
		Positive regulator of OL differentiation in En1-area			[16]
		Negative regulator of apoptosis			[3,16]
	**Sox10**	Regulated by Zfp24			[39]
		Regulated by Olig2			[38]
		Regulated by Chd7			[57]
		Affects OPC identity	Ascl1, Olig2 + E47	*Cspg4*	[6,9,15]
		Positive regulator of OPC proliferation, migration and survival		*Pdgfra*	[17]
		Positive regulator of OL differentiation		*miR-335, miR-338*	[35]
				*Mbp*	[36]
				*Mog*	[50]
				*Nfatc2*	[45]
				*Tcf7l2*	[44]
			Chd7	*Plp1, Cnp1*	[56,57]
			Tcf7l2	*Mature myelin and cholesterol synthesis genes*	[41]
			Ep400	*Myrf*	[48]
			Myrf	*Dusp15*	[40]
			Nfatc2	*Nkx2.2*	[45]
			Zfp24	*Myrf*	[37,39]
		Negatively regulates transient Tcf7l2 expression		*miR-155, miR-338*	[44]
		Positive regulator of remyelination	Chd7	*Myrf, Nkx2.2, Mbp*	[56]
		Positive regulator of OL myelin maintenance			[50]
**SoxF**	**Sox17**	Positive regulator of OL differentiation		Wnt signaling	[31,32,33]
		Negative regulator of proliferation		*cyclinD1*	[31,32]
		Negative regulator of OL differentiation			[34]

## Abbreviations

OL	oligodendrocytes
CNS	central nervous system
OPC	oligodendrocyte precursor cell
NPC	neural precursor cell
HMG	high mobility group
E	embryonic day
P	postnatal day
hPSC	human pluripotent stem cell
ChIP	chromatin immunoprecipitation
EMSA	electromobility shift assay
vr1	ventral rhombomere 1
NSC	neural stem cell

## References

[1] Küspert M., Wegner M. (2016). SomethiNG 2 talk about-Transcriptional regulation in embryonic and adult oligodendrocyte precursors. Brain Res..

[2] Reiprich S., Wegner M. (2015). From CNS stem cells to neurons and glia: Sox for everyone. Cell Tissue Res..

[3] Stolt C.C., Lommes P., Sock E., Chaboissier M.C., Schedl A., Wegner M. (2003). The Sox9 transcription factor determines glial fate choice in the developing spinal cord. Genes Dev..

[4] Stolt C.C., Lommes P., Friedrich R.P., Wegner M. (2004). Transcription factors Sox8 and Sox10 perform non-equivalent roles during oligodendrocyte development despite functional redundancy. Development.

[5] Stolt C.C., Schmitt S., Lommes P., Sock E., Wegner M. (2005). Impact of transcription factor Sox8 on oligodendrocyte specification in the mouse embryonic spinal cord. Dev. Biol..

[6] Garcia-Leon J.A., Kumar M., Boon R., Chau D., One J., Wolfs E., Eggermont K., Berckmans P., Gunhanlar N., de Vrij F. (2018). SOX10 Single Transcription Factor-Based Fast and Efficient Generation of Oligodendrocytes from Human Pluripotent Stem Cells. Stem Cell Rep..

[7] Yang N., Zuchero J.B., Ahlenius H., Marro S., Ng Y.H., Vierbuchen T., Hawkins J.S., Geissler R., Barres B.A., Wernig M. (2013). Generation of oligodendroglial cells by direct lineage conversion. Nat. Biotechnol..

[8] Najm F.J., Lager A.M., Zaremba A., Wyatt K., Caprariello A.V., Factor D.C., Karl R.T., Maeda T., Miller R.H., Tesar P.J. (2013). Transcription factor-mediated reprogramming of fibroblasts to expandable, myelinogenic oligodendrocyte progenitor cells. Nat. Biotechnol..

[9] Gotoh H., Wood W.M., Patel K.D., Factor D.C., Boshans L.L., Nomura T., Tesar P.J., Ono K., Nishiyama A. (2018). NG2 expression in NG2 glia is regulated by binding of SoxE and bHLH transcription factors to a Cspg4 intronic enhancer. Glia.

[10] Tomassy G.S., Berger D.R., Chen H.H., Kasthuri N., Hayworth K.J., Vercelli A., Seung H.S., Lichtman J.W., Arlotta P. (2014). Distinct profiles of myelin distribution along single axons of pyramidal neurons in the neocortex. Science.

[11] Biname F. (2014). Transduction of extracellular cues into cell polarity: The role of the transmembrane proteoglycan NG2. Mol. Neurobiol..

[12] Stallcup W.B., Huang F.J. (2008). A role for the NG2 proteoglycan in glioma progression. Cell Adh. Migr..

[13] Scott C.E., Wynn S.L., Sesay A., Cruz C., Cheung M., Gomez Gaviro M.V., Booth S., Gao B., Cheah K.S., Lovell-Badge R. (2010). SOX9 induces and maintains neural stem cells. Nat. Neurosci..

[14] Bergsland M., Ramskold D., Zaouter C., Klum S., Sandberg R., Muhr J. (2011). Sequentially acting Sox transcription factors in neural lineage development. Genes Dev..

[15] Klum S., Zaouter C., Alekseenko Z., Bjorklund A.K., Hagey D.W., Ericson J., Muhr J., Bergsland M. (2018). Sequentially acting SOX proteins orchestrate astrocyte- and oligodendrocyte-specific gene expression. EMBO Rep..

[16] Hashimoto R., Hori K., Owa T., Miyashita S., Dewa K., Masuyama N., Sakai K., Hayase Y., Seto Y., Inoue Y.U. (2016). Origins of oligodendrocytes in the cerebellum, whose development is controlled by the transcription factor, Sox9. Mech. Dev..

[17] Finzsch M., Stolt C.C., Lommes P., Wegner M. (2008). Sox9 and Sox10 influence survival and migration of oligodendrocyte precursors in the spinal cord by regulating PDGF receptor alpha expression. Development.

[18] Stolt C.C., Schlierf A., Lommes P., Hillgärtner S., Werner T., Kosian T., Sock E., Kessaris N., Richardson W.D., Lefebvre V. (2006). SoxD proteins influence multiple stages of oligodendrocyte development and modulate SoxE protein function. Dev. Cell.

[19] Baroti T., Zimmermann Y., Schillinger A., Liu L., Lommes P., Wegner M., Stolt C.C. (2016). Transcription factors Sox5 and Sox6 exert direct and indirect influences on oligodendroglial migration in spinal cord and forebrain. Glia.

[20] Baroti T., Schillinger A., Wegner M., Stolt C.C. (2016). Sox13 functionally complements the related Sox5 and Sox6 as important developmental modulators in mouse spinal cord oligodendrocytes. J. Neurochem..

[21] Potzner M.R., Griffel C., Lutjen-Drecoll E., Bösl M.R., Wegner M., Sock E. (2007). Prolonged Sox4 expression in oligodendrocytes interferes with normal myelination in the central nervous system. Mol. Cell Biol..

[22] Braccioli L., Vervoort S.J., Puma G., Nijboer C.H., Coffer P.J. (2018). SOX4 inhibits oligodendrocyte differentiation of embryonic neural stem cells in vitro by inducing Hes5 expression. Stem Cell Res..

[23] Kondo T., Raff M. (2000). Basic helix-loop-helix proteins and the timing of oligodendrocyte differentiation. Development.

[24] Liu A., Li J., Marin-Husstege M., Kageyama R., Fan Y., Gelinas C., Casaccia-Bonnefil P. (2006). A molecular insight of Hes5-dependent inhibition of myelin gene expression: Old partners and new players. EMBO J..

[25] Graham V., Khudyakov J., Ellis P., Pevny L. (2003). SOX2 Functions to Maintain Neural Progenitor Identity. Neuron.

[26] Masui S., Nakatake Y., Toyooka Y., Shimosato D., Yagi R., Takahashi K., Okochi H., Okuda A., Matoba R., Sharov A.A. (2007). Pluripotency governed by Sox2 via regulation of Oct3/4 expression in mouse embryonic stem cells. Nat. Cell Biol..

[27] Bylund M., Andersson E., Novitch B.G., Muhr J. (2003). Vertebrate neurogenesis is counteracted by Sox1-3 activity. Nat. Neurosci..

[28] Hoffmann S.A., Hos D., Küspert M., Lang R.A., Lovell-Badge R., Wegner M., Reiprich S. (2014). Stem cell factor Sox2 and its close relative Sox3 have differentiation functions in oligodendrocytes. Development.

[29] Zhang S., Rasai A., Wang Y., Xu J., Bannerman P., Erol D., Tsegaye D., Wang A., Soulika A., Zhan X. (2018). The Stem Cell Factor Sox2 Is a Positive Timer of Oligodendrocyte Development in the Postnatal Murine Spinal Cord. Mol. Neurobiol..

[30] Zhang S., Zhu X., Gui X., Croteau C., Song L., Xu J., Wang A., Bannerman P., Guo F. (2018). Sox2 Is Essential for Oligodendroglial Proliferation and Differentiation during Postnatal Brain Myelination and CNS Remyelination. J. Neurosci..

[31] Chew L.J., Shen W., Ming X., Senatorov V.V., Chen H.L., Cheng Y., Hong E., Knoblach S., Gallo V. (2011). SRY-box containing gene 17 regulates the Wnt/beta-catenin signaling pathway in oligodendrocyte progenitor cells. J. Neurosci..

[32] Sohn J., Natale J., Chew L.J., Belachew S., Cheng Y., Aguirre A., Lytle J., Nait-Oumesmar B., Kerninon C., Kanai-Azuma M. (2006). Identification of Sox17 as a transcription factor that regulates oligodendrocyte development. J. Neurosci..

[33] Ming X., Chew L.J., Gallo V. (2013). Transgenic overexpression of Sox17 promotes oligodendrocyte development and attenuates demyelination. J. Neurosci..

[34] Fauveau M., Wilmet B., Deboux C., Benardais K., Bachelin C., Temporao A.C., Kerninon C., Nait Oumesmar B. (2018). SOX17 transcription factor negatively regulates oligodendrocyte precursor cell differentiation. Glia.

[35] Reiprich S., Cantone M., Weider M., Baroti T., Wittstatt J., Schmitt C., Küspert M., Vera J., Wegner M. (2017). Transcription factor Sox10 regulates oligodendroglial Sox9 levels via microRNAs. Glia.

[36] Stolt C.C., Rehberg S., Ader M., Lommes P., Riethmacher D., Schachner M., Bartsch U., Wegner M. (2002). Terminal differentiation of myelin-forming oligodendrocytes depends on the transcription factor Sox10. Genes Dev..

[37] Hornig J., Fröb F., Vogl M.R., Hermans-Borgmeyer I., Tamm E.R., Wegner M. (2013). The transcription factors Sox10 and Myrf define an essential regulatory network module in differentiating oligodendrocytes. PLoS Genet..

[38] Küspert M., Hammer A., Bösl M.R., Wegner M. (2011). Olig2 regulates Sox10 expression in oligodendrocyte precursors through an evolutionary conserved distal enhancer. Nucleic Acids Res..

[39] Elbaz B., Aaker J.D., Isaac S., Kolarzyk A., Brugarolas P., Eden A., Popko B. (2018). Phosphorylation State of ZFP24 Controls Oligodendrocyte Differentiation. Cell Rep..

[40] Muth K.N., Piefke S., Weider M., Sock E., Hermans-Borgmeyer I., Wegner M., Küspert M. (2016). The Dual-specificity phosphatase Dusp15 is regulated by Sox10 and Myrf in Myelinating Oligodendrocytes. Glia.

[41] Zhao C., Deng Y., Liu L., Yu K., Zhang L., Wang H., He X., Wang J., Lu C., Wu L.N. (2016). Dual regulatory switch through interactions of Tcf7l2/Tcf4 with stage-specific partners propels oligodendroglial maturation. Nat. Commun..

[42] Fancy S.P., Baranzini S.E., Zhao C., Yuk D.I., Irvine K.A., Kaing S., Sanai N., Franklin R.J., Rowitch D.H. (2009). Dysregulation of the Wnt pathway inhibits timely myelination and remyelination in the mammalian CNS. Genes Dev..

[43] Ye F., Chen Y., Hoang T., Montgomery R.L., Zhao X.H., Bu H., Hu T., Taketo M.M., van Es J.H., Clevers H. (2009). HDAC1 and HDAC2 regulate oligodendrocyte differentiation by disrupting the beta-catenin-TCF interaction. Nat. Neurosci..

[44] Cantone M., Küspert M., Reiprich S., Lai X., Eberhardt M., Göttle P., Beyer F., Azim K., Küry P., Wegner M. (2019). A gene regulatory architecture that controls region-independent dynamics of oligodendrocyte differentiation. Glia.

[45] Weider M., Starost L.J., Groll K., Küspert M., Sock E., Wedel M., Fröb F., Schmitt C., Baroti T., Hartwig A.C. (2018). Nfat/calcineurin signaling promotes oligodendrocyte differentiation and myelination by transcription factor network tuning. Nat. Commun..

[46] Hogan P.G., Chen L., Nardone J., Rao A. (2003). Transcriptional regulation by calcium, calcineurin, and NFAT. Genes Dev..

[47] Cheli V.T., Santiago Gonzalez D.A., Spreuer V., Paez P.M. (2015). Voltage-gated Ca2+ entry promotes oligodendrocyte progenitor cell maturation and myelination in vitro. Exp. Neurol..

[48] Elsesser O., Fröb F., Küspert M., Tamm E.R., Fujii T., Fukunaga R., Wegner M. (2019). Chromatin remodeler Ep400 ensures oligodendrocyte survival and is required for myelination in the vertebrate central nervous system. Nucleic Acids Res..

[49] Kellerer S., Schreiner S., Stolt C.C., Scholz S., Bösl M.R., Wegner M. (2006). Replacement of the Sox10 transcription factor by Sox8 reveals incomplete functional equivalence. Development.

[50] Turnescu T., Arter J., Reiprich S., Tamm E.R., Waisman A., Wegner M. (2018). Sox8 and Sox10 jointly maintain myelin gene expression in oligodendrocytes. Glia.

[51] Touraine R.L., Attie-Bitach T., Manceau E., Korsch E., Sarda P., Pingault V., Encha-Razavi F., Pelet A., Auge J., Nivelon-Chevallier A. (2000). Neurological phenotype in Waardenburg syndrome type 4 correlates with novel SOX10 truncating mutations and expression in developing brain. Am. J. Hum. Genet..

[52] Feng W., Khan M.A., Bellvis P., Zhu Z., Bernhardt O., Herold-Mende C., Liu H.K. (2013). The chromatin remodeler CHD7 regulates adult neurogenesis via activation of SoxC transcription factors. Cell Stem Cell.

[53] Sanlaville D., Etchevers H.C., Gonzales M., Martinovic J., Clement-Ziza M., Delezoide A.L., Aubry M.C., Pelet A., Chemouny S., Cruaud C. (2006). Phenotypic spectrum of CHARGE syndrome in fetuses with CHD7 truncating mutations correlates with expression during human development. J. Med. Genet..

[54] Jongmans M.C., Admiraal R.J., van der Donk K.P., Vissers L.E., Baas A.F., Kapusta L., van Hagen J.M., Donnai D., de Ravel T.J., Veltman J.A. (2006). CHARGE syndrome: The phenotypic spectrum of mutations in the CHD7 gene. J. Med. Genet..

[55] Feng W., Kawauchi D., Korkel-Qu H., Deng H., Serger E., Sieber L., Lieberman J.A., Jimeno-Gonzalez S., Lambo S., Hanna B.S. (2017). Chd7 is indispensable for mammalian brain development through activation of a neuronal differentiation programme. Nat. Commun..

[56] He D., Marie C., Zhao C., Kim B., Wang J., Deng Y., Clavairoly A., Frah M., Wang H., He X. (2016). Chd7 cooperates with Sox10 and regulates the onset of CNS myelination and remyelination. Nat. Neurosci..

[57] Marie C., Clavairoly A., Frah M., Hmidan H., Yan J., Zhao C., Van Steenwinckel J., Daveau R., Zalc B., Hassan B. (2018). Oligodendrocyte precursor survival and differentiation requires chromatin remodeling by Chd7 and Chd8. Proc. Natl Acad Sci USA.

[58] Doi T., Ogata T., Yamauchi J., Sawada Y., Tanaka S., Nagao M. (2017). Chd7 Collaborates with Sox2 to Regulate Activation of Oligodendrocyte Precursor Cells after Spinal Cord Injury. J. Neurosci..

